# Editorial: The diagnoses of glaucoma in the era of artificial intelligence

**DOI:** 10.3389/fopht.2024.1496533

**Published:** 2024-12-18

**Authors:** Saif Aldeen AlRyalat, Muawyah Al Bdour, Hisham M. Jammal

**Affiliations:** ^1^ Department of Ophthalmology, University of Colorado Anschutz Medical Campus, Aurora, CO, United States; ^2^ Department of Ophthalmology, The University of Jordan, Amman, Jordan; ^3^ Department of Ophthalmology, Jordan University of Science and Technology, Irbid, Jordan

**Keywords:** glaucoma, artificial intelligence, deep learning, large language model (LLM), ChatGPT

## Introduction

The success of any artificial intelligence (AI) project, particularly in the medical field, is heavily reliant on the quality and comprehensiveness of the data used to train the model. Recent years have witnessed remarkable progress in AI development, especially with the emergence of large language models and other advanced techniques that have revolutionized data processing and analysis. Despite these technological advances, a critical challenge persists in ophthalmology, particularly in the realm of glaucoma diagnosis, where the availability of large, well-curated datasets is limited. In contrast to other medical specialties where extensive, openly accessible datasets are readily available for research (1), the field of ophthalmology suffers from a scarcity of comprehensive, structured datasets. The few datasets that do exist are often fragmented, with incomplete metadata and insufficient standardization, making it difficult to conduct robust and reproducible AI studies. This Research Topic is particularly pronounced in glaucoma research, where even the “ground truth” is not universally agreed upon. The absence of a well-defined diagnostic standard in glaucoma adds a layer of complexity to AI-based studies. As different research groups may rely on varying criteria to establish a diagnosis, AI models trained on disparate “ground truths” can produce misleading results. High accuracy reported in such studies may not necessarily reflect the AI’s true diagnostic capability but rather the inconsistency or unreliability of the data it was trained on. This highlights a significant limitation in current glaucoma research, where a model’s performance might be inflated due to flawed or poorly defined reference standards (2). One of the key articles in this Research Topic delves into this Research Topic, examining the implications of inconsistent ground truths in AI studies on glaucoma and exploring various other critical concepts relevant to AI-assisted glaucoma diagnosis (AlShawabkeh et al.). We also explored the use of large language models in glaucoma diagnosis, where previous articles proved its effectiveness in other domains (3–5). As we look toward the future, it becomes imperative to address these data-related challenges to enable the development of AI models that can truly transform the way glaucoma is diagnosed.

## Contributions

The articles in this Research Topic addressed critical challenges related to AI in glaucoma, such as the acquisition of reliable data, the development of AI models, and the standardization of ground truth in glaucoma diagnosis. The development of AI models and the challenge of defining a “ground truth” in glaucoma diagnosis is tackled in two other contributions. The article *“The Utilization of Artificial Intelligence in Glaucoma: Diagnosis versus Screening”* (AlShawabkeh et al.), discusses the unique roles AI can play in both screening and diagnostic settings, each with different performance criteria. Screening models prioritize sensitivity, aiming to capture as many potential glaucoma cases as possible, while diagnostic models emphasize specificity to accurately confirm the disease. The balance between sensitivity and specificity, particularly in AI models built from multimodal data (such as fundus photographs and functional tests), is critical for ensuring that AI applications can distinguish glaucomatous optic neuropathy with precision. The article *“Evaluating the Strengths and Limitations of Multimodal Chat GPT-4 in Detecting Glaucoma Using Fundus Images”* (AlRyalat et al.), explores the potential of advanced AI models, specifically large language models like ChatGPT-4, in the field of glaucoma diagnosis. Despite no prior fine-tuning, the model demonstrated a relatively acceptable diagnostic accuracy using fundus images, underscoring the versatility and power of multimodal AI. This work points toward a future where generative and multimodal AI models might reduce the need for extensive data, streamlining the development of diagnostic tools that could be deployed in resource-constrained environments.

Two of the contributions focus on essential data related to glaucoma diagnosis, highlighting the importance of accurate intraocular pressure (IOP) measurements and imaging data, both of which are crucial for AI model training. In the article, *“Evaluation of agreement of IOP measurements by Tono-Vera Tonometer to Goldmann Applanation Tonometry,”* (Niles et al.), the authors validate the Tono-Vera tonometer as a reliable tool for measuring IOP in glaucoma patients. Similarly, the article *“Pilot Report: Objective Quantification of Trabecular Meshwork Pigmentation Using Densitometry and the NIDEK GS1 Gonioscope in Glaucoma Patients”* (Laroche et al.), introduces a novel methodology to quantify trabecular meshwork pigmentation, a potential biomarker for early detection of pigmentary glaucoma. This work provides valuable insights into how image-based biomarkers could serve as critical data sources for future AI applications in glaucoma, emphasizing the need for rich and diverse imaging datasets in the field.

## Future directions

In a previous article, we proposed that we must “go back to the basics” and establish a definitive understanding of how to diagnose glaucoma ourselves before attempting to train AI models for the same purpose (6). This idea stemmed from another work that reviewed the diagnostic criteria used in landmark clinical trials for glaucoma, where, notably, no universally agreed-upon definition of the disease was found (7). The lack of a gold standard for glaucoma diagnosis complicates the training of AI models, as inconsistencies in the ground truth limit the ability of models to learn effectively. A more definitive, albeit non-practical, approach would be to focus on cases where disease progression is clearly documented with correlated structural defects and where other etiologies, such as neurologic or retinal diseases, have been rigorously ruled out. Looking forward, one promising approach would be to reverse the traditional AI training strategy. Rather than starting with ambiguous or mixed datasets, we could first focus on what we know with high certainty: cases where visual field defects are clearly not due to glaucoma. This includes conditions like neurological visual field defects, artifacts, or retinal diseases. By training AI models on these negative examples—instances where glaucoma has been definitively ruled out—we can better define the boundaries of non-glaucomatous cases. These models can serve as a filtering mechanism, reducing false positives and enabling more accurate screening of glaucoma suspects. We know from previous prevalence studies that reliable field defects can present in up to one fifth of elderly population, with etiologies including glaucoma in approximately third of cases, with the rest being neurological or retinal in etiology (8, 9). Combining these two approaches—training AI on both the absence and presence of glaucoma—could yield models that are not only more accurate but also better equipped to handle the complexity of real-world diagnosis.
